# Mn–Fe dual metal–organic framework based on trimesic acid as a high-performance electrode for lithium metal batteries†

**DOI:** 10.1039/d4na00600c

**Published:** 2024-09-24

**Authors:** Saira Sarwar, Verónica Montes-García, Maria Stachowiak, Tomasz Chudziak, Wojciech Kukułka, Cataldo Valentini, Krzysztof Karoń, Dawid Pakulski, Artur Ciesielski

**Affiliations:** a Center for Advanced Technologies, Adam Mickiewicz University Uniwersytetu Poznańskiego 10 Poznań 61-614 Poland dawid.pakulski@amu.edu.pl; b Université de Strasbourg, CNRS ISIS 8 allée Gaspard Monge Strasbourg 67000 France montesgarcia@unistra.fr ciesielski@unistra.fr; c Faculty of Chemistry, Silesian University of Technology Strzody 9 44-100 Gliwice Poland; d Faculty of Chemistry, Adam Mickiewicz University Uniwersytetu Poznańskiego 8 Poznań 61-614 Poland

## Abstract

A novel Mn–Fe dual metal–organic framework (Mn-Fe-BTC DMOF) was synthesized *via* a one-step hydrothermal method and employed as a cathode material in lithium metal batteries. The Mn-Fe-BTC DMOF exhibited a high initial capacity (1385 mA h g^−1^) and after 100 cycles (687 mA h g^−1^), demonstrating its potential for high-performance energy storage devices.

## Abstract 2

A novel dual metal–organic framework (Mn-Fe-BTC DMOF) was synthesized, demonstrating a promising strategy for improving MOF's surface area, chemical stability, and electrochemical performance in lithium metal batteries.

## Graphical abstract

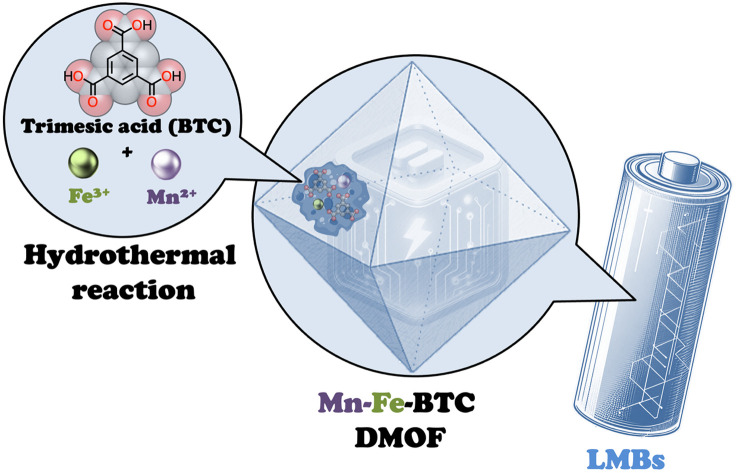

The risks of resource depletion, environmental pollution, and political instability have accelerated the use of renewable energy sources such as tidal power, wind, and solar energy. To effectively integrate these renewable solutions into the power grid, large-scale energy storage systems (ESS) are necessary for optimal operation.^1–3^ Lithium-ion batteries (LIBs) are essential for modern technology because of their high energy density, long cycle life, and low self-discharge. Among the latest generations of LIBs, lithium metal batteries (LMBs), which use lithium metal as the negative electrode, offer even higher energy densities and improved performance, potentially extending the applications of LMBs and enhancing device efficiency. LMBs commonly utilize various cathode materials based on transition metal oxides, including lithium cobalt oxide (LiCoO_2_),^4^ lithium iron phosphate (LiFePO_4_),^5^ lithium manganese oxide (LiMn_2_O_4_),^6^ nickel manganese cobalt oxide (NMC),^7^ nickel cobalt aluminum oxide (NCA)^8^ and lithium nickel oxide (LiNiO_2_)^9^ due to their superior theoretical capacities. However, the use of each of these cathodes still faces challenges, including high cost, stability issues, lower energy density, capacity fading, or safety concerns. These challenges have spurred research into novel and sustainable cathode materials.

Metal–organic frameworks (MOFs), comprising metal ions and organic ligands, are a class of porous organic materials that offer promising advantages as cathode materials for LMBs due to their high surface area, tunable pore structure, and customizable functionality.^10^ Their porous nature facilitates efficient lithium-ion diffusion, potentially enhancing battery performance.^11^ Tarascon *et al.* were the first to introduce a Fe-based MOF (MIL-53 MOF) as a cathode in LIBs, displaying a modest capacity of 75 mA h g^−1^. Since then, a variety of MOFs have been explored as potential cathode or anode materials. Although some encouraging progress has been made, challenges persist, including low capacity, limited electrical conductivity, and stability issues during cycling, hindering the wider application of MOFs in LMBs.

To comprehend these drawbacks, it is essential to understand the lithium-ion storage mechanisms. The two primary lithium storage mechanisms are intercalation/insertion and conversion. Intercalation often yields low capacity, whereas conversion, typically reliant on the redox activity of MOFs' central metal ions, often leads to poor cycling stability. To address the cyclability challenge associated with the conversion mechanism in MOFs, various strategies can be pursued. For instance, MOFs can be engineered to facilitate redox processes exclusively within the organic linkers. This approach enables the combination of inorganic cluster rigidity with organic ligand flexibility, potentially preserving the MOF's three-dimensional (3D) framework during cycling. Moreover, the use of organic linkers with aromatic cores might enhance π–π interactions, thereby reinforcing the chemical stability of the MOF's 3D structure.

Trimesic acid (*i.e.*, 1,3,5-benzenetricarboxylic acid, BTC)-based MOFs, whose electrochemical properties stem from interactions with Li^+^ ions mediated by redox-active carbonyl groups, have exhibited remarkable performance. Metal–organic frameworks with single metal ions (M) have been studied thoroughly in the literature, in the fabrication of M-BTC MOF-based electrodes for LMBs, including Cu,^12^ Ni,^13^ Mn^14^ and Fe,^15^ achieving capacities of 740, 1085, 694, and 1021 mA h g^−1^, respectively. Among these single metals, Fe-BTC is notable for its higher specific surface area (1125 m^2^ g^−1^) compared to Cu-BTC (489.4 m^2^ g^−1^), Ni-BTC (551 m^2^ g^−1^), and Mn-BTC (23.8 m^2^ g^−1^). Interestingly, despite its smallest surface area, Mn-BTC maintains nearly constant cycling stability over 100 charge/discharge cycles. In contrast, the other M-BTC MOFs show increased capacity upon cycling, indicating irreversible structural changes. Recently, Zheng *et al.* proposed an innovative strategy to enhance the electrochemical performance of MOF cathodes in LMBs.^16^ Using BTC as the organic linker and incorporating two metal ions, Mn and Co (Mn-Co-BTC), this dual metal–organic framework (DMOF) achieved a superior capacity of 819 mA h g^−1^, surpassing that of the previously reported Mn-BTC MOF. Additionally, Mn-Co-BTC DMOF maintained a significant lithium storage capacity of 337 mA h g^−1^ after 600 galvanostatic charge/discharge cycles at 1 A g^−1^. Incorporating two metal ions enhances Li^+^ ion transport and improves structural stability, boosting lithium storage capabilities – a promising but barely underexplored strategy in MOFs. Given Fe-BTC's high electrochemical performance and large surface area, along with Mn-BTC's chemical stability, we foresee that a DMOF combining BTC as the organic linker and Mn(ii) and Fe(iii) as metal ions (Mn-Fe-BTC) would lead to enhanced electrochemical performance in LMBs (Fig. 1 and S1†).

**Fig. 1:**
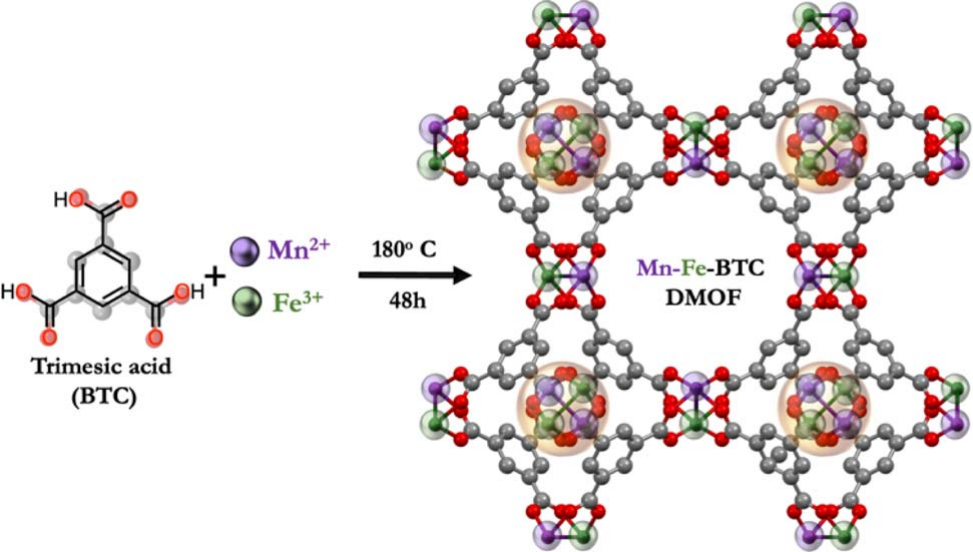
Dual metal–organic framework as electrode for LMBs.

Mn-Fe-BTC DMOF is synthesized using a modified version of a previously reported protocol.^17^ A hydrothermal process is employed using a mixture of manganese chloride and iron chloride in ethanol at 180 °C for 48 hours (see ESI† for details). The chemical structure of Mn-Fe-BTC DMOF is first confirmed by Raman spectroscopy (Fig. S2†). The Raman spectrum of Mn-Fe-BTC DMOF shows the characteristic peak of BTC at 998 (C=C), 1005 cm^−1^.^18^ The formation of Mn-Fe-BTC DMOF is proven by the appearance of a new peak at 495 cm^−1^, corresponding to the metal–oxygen interactions.^19–21^ Furthermore, the vibrations at 381 cm^−1^ (O–C=O), 1332 cm^−1^ (C–O) and 1653 cm^−1^ (C=O) of BTC are shifted to 293, 1375 and 1612 cm^−1^, respectively, in Mn-Fe-BTC DMOF due to the metal-carboxylate bonding. The Fourier-transform infrared spectroscopy (FTIR) spectrum of BTC (Fig. S3†), displays peaks at 1694 and 1262 cm^−1^ attributed to asymmetric and symmetric vibrations of the carboxylate groups and a peak at 738 cm^−1^, assigned to the C–H vibration of the benzene ring. In agreement with the Raman analysis, in the FTIR spectrum of Mn-Fe-BTC DMOF, the asymmetric and symmetric vibrations of the carboxylate groups are shifted to 1627 and 1381 cm^−1^, respectively, due to their interactions with the metal ions. While Raman and FTIR analyses prove the presence of metal–oxygen interactions, X-ray photoelectron spectroscopy (XPS) analysis is used to shed light on the nature of the metal ion (Fig. S4†). On the one hand, the XPS spectrum of Fe 2p exhibits two characteristic main peaks of Fe^3+^ at binding energies of 711.6 eV (Fe 2p_3/2_) and 724.8 eV (Fe 2p_1/2_). On the other hand, the spectrum of Mn 2p displays two characteristic main peaks of Mn^2+^ at binding energies of 642.2 eV (Mn 2p_3/2_) and of 644.9 eV (Mn 2p_1/2_).^22^ Therefore XPS unequivocally proves the presence of both metal ions in the organic framework. The thermal stability of Mn-Fe-BTC is investigated through thermogravimetric analysis (TGA) (Fig. S5†). The thermogram of Mn-Fe-BTC exhibits remarkable thermal stability, with Td_10_ values (thermal decomposition of 10% weight) of approximately 310 °C. Notably, the thermal decomposition of Mn-Fe-BTC DMOF occurs above 350 °C, which is attributed to the decomposition of the BTC.^23^

The morphology and microstructure of Mn-Fe-BTC DMOF are evaluated using scanning electron microscopy (SEM) (Fig. 2a and S6†) and N_2_ adsorption/desorption isotherm analysis (Fig. 2b and c), respectively. The analysis reveals that Mn-Fe-BTC primarily consists of micrometer-sized cubic structures. Brunauer–Emmett–Teller (BET) model is used for the calculation of the specific surface area (SSA) and Density Functional Theory (DFT) is used for the determination of the pore size distribution. The Mn-Fe-BTC DMOF sample exhibits a SSA of 1045 m^2^ g^−1^ and pores with an average diameter of 1.6 nm. The SSA of Mn-Fe-BTC is slightly lower than the one of Fe-BTC (1125 m^2^ g^−1^)^15^ although much higher than the one of Mn-BTC (23.8 m^2^ g^−1^),^14^ indicating that the presence of Mn in the dual MOF does not have a significant effect on lowering the SSA value. Furthermore, the isotherms exhibit an H3 type hysteresis loop, indicating the presence of loosely assembled particles forming slit-like pores.^24^ The crystalline structure of Mn-Fe-BTC is analyzed by powder X-ray diffraction (PXRD) (Fig. 2d). The peaks observed at 9.4, 9.9, 17.6, 19.2, 26.5, and 31.7° for Mn-Fe-BTC are in good agreement with the earlier reported Mn-Fe-MOF based on terephthalic acid as a linker.^25,26^ Moreover, the prominent peak at 2*θ* = 10.7° presented on pristine BTC is absent of Mn-Fe-BTC, additionally confirming the formation of the crystalline framework.

**Fig. 2:**
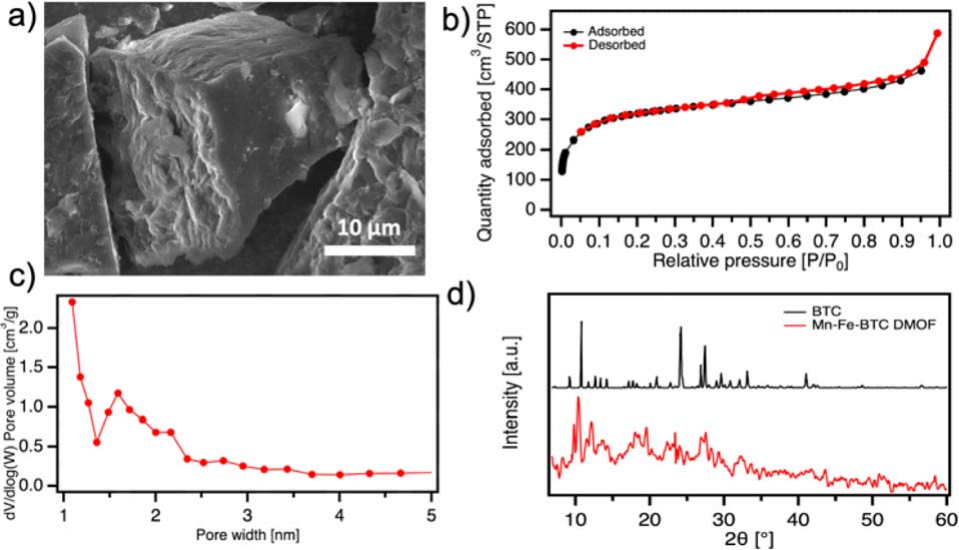
(a) SEM image of Mn-Fe-BTC DMOF, (b) N_2_ isotherm adsorption/desorption isotherm analysis of Mn-Fe-BTC DMOF, (c) pore size distribution of Mn-Fe-BTC DMOF and (d) PXRD spectra of BTC (black) and Mn-Fe-BTC DMOF (red).

The electrochemical performance of DMOF active material *versus* Li/Li^+^ is investigated by cyclic voltammetry (CV), galvanostatic charge–discharge (GCD) and electrochemical impedance spectroscopy (EIS) using coin cells (CR2032) with Mn-Fe-BTC DMOF and Li metal as a cathode and anode, respectively. Fig. S7† displays the first two CV scans of Mn-Fe-BTC DMOF electrode in the potential window of 0.01–3.0 V. During the first discharging cycle, three peaks are observed at ∼1.37, 0.99 and 0.63 V, respectively. The broad small peak at 1.37 V could be associated with an irreversible reaction related to the formation of solid electrolyte interface (SEI) layers due to electrolyte decomposition.^27,28^ Differently, the peak at around 0.99 V may be attributed to the reduction of manganese ions.^12^ The last broad peak centered at 0.59 V might be attributed to the reduction of Fe^3+^ to Fe^2+^ as well as the insertion of Li^+^ ion to the organic framework.^15,29,30^ The corresponding anodic broad peaks, centered at ∼0.85, 1.50 and 1.85 V, respectively, indicate the Li extraction from the aromatic C_6_ ring/carboxylate moieties as well as the reversible oxidation of the metal ions. The broad anodic peaks possibly indicate a gradual, multistep process for lithium extraction.^14,29^

The electrochemical performance from GCD of the Mn-Fe-BTC electrode is assessed at various current densities from 0.1 to 2 A g^−1^, as illustrated in Fig. 3b. The reversible specific charge–discharge capacities are measured at 0.1, 0.2, 0.5, 1 and 2 A g^−1^ and amount to 1292/1385, 962/1016, 735/792, 597/640, 496/537 mA h g^−1^, respectively. Mn-Fe-BTC exhibits significantly higher specific capacity at high current densities (597/640 mA h g^−1^ at 1 A g^−1^ and 496/537 mA h g^−1^ at 2 A g^−1^) compared to mono- Mn-BTC (435/433 mA h g^−1^ at 1 A g^−1^),^28^ Fe-BTC (302/304 mA h g^−1^ at 2 A g^−1^)^15^ and bimetallic MOF with BTC linkers Mn-Co-BTC MOFs (445 mA h g^−1^ at 0.5 A g^−1^) and other MOF-based materials (Table S1†).^31^ More importantly, a high specific capacity of around 1050 mA h g^−1^ is recovered when the current density is reduced from 2 to 0.1 A g^−1^, demonstrating the robustness of the DMOF structure. The initial discharge capacity of the Fe-Mn-BTC DMOF electrode is above 1000 mA h g^−1^ (Fig. S8†), but it decreases to 765 mA h g^−1^ after 10 cycles. However, after approximately fifty cycles, the Fe-Mn-BTC DMOF electrode exhibits a stable capacity for reversible lithium storage of about 710 mA h g^−1^, indicating good cycling stability (Fig. 3d and S8†). Additionally, we observed that the charge–discharge plateaus shift regularly during subsequent cycles, likely due to the increase in resistance and the SEI formation. Electrochemical impedance spectroscopy (EIS) data is analyzed using Nyquist plots (Fig. 3c) and the experimental results are well-fitted with the indicated circuit (Fig. S9†). DMOF material exhibits a low charge transfer resistance (*R*_ct_) of *R*_ct_ = 60 Ω indicating that dual MOFs allow for efficient and rapid transfer of charge (electrons or ions) in the electrode's surface Another appealing feature of the novel Mn-Fe-BTC DMOF electrode is its robust long-term cyclability at a rate of 200 mA g^−1^, as demonstrated in Fig. 3d. After the first three cycles, the coulombic efficiency of the Mn-Fe-BTC DMOF rapidly increased to 99.9% and remains around 100% throughout the subsequent cycles. After 100 charge–discharge cycles, a high specific capacity of 687 mA h g^−1^ is still obtained for Mn-Fe-BTC DMOF. The high chemical stability of DMOF electrode can be attributed to the presence of conjugated carboxylates, which contain aromatic cores with strong π–π interactions. In order to determine the chemical stability of Mn-Fe-BTC DMOF a series of *ex situ* characterizations are performed (Fig. S10–12†). Fig. S10† displays the high-resolution spectra of Fe 2p, Mn 2p,and C 1s for Mn-Fe-BTC DMOF. Notably, the XPS spectra exhibit no significant changes compared to the pristine state for any of the elements, demonstrating the DMOF's exceptional chemical stability during electrochemical cycling. Similarly, the XRD analysis (Fig. S11†) shows consistent peaks with no shifts, confirming the structural integrity of the active material. Additionally, SEM analysis (Fig. S12†) after cycling reveals that the structure remains porous and that the pores have not collapsed. EIS and CV characterizations after 100 charge–discharge cycles are presented in Fig. S13 and 14.† The Nyquist plots for Mn-Fe-BTC DMOF (Fig. S13†) after 1 cycle (black) and 100 cycles (red) reveal an increase in *R*_ct_ from 60 Ω to 189 Ω, indicating the formation of a SEI on the electrode surface. As shown in Fig. 3d, after a few cycles, the specific capacity becomes constant, suggesting that the SEI layer has reached a stable state.^32,33^ Importantly, no additional semicircles are observed, confirming the material's stability.^34^ The CV results support the EIS findings, showing that the increased resistance from the SEI formation, slows the charge transfer process, leading to a reduction in the peak heights and overall CV area (Fig. S14†).

**Fig. 3:**
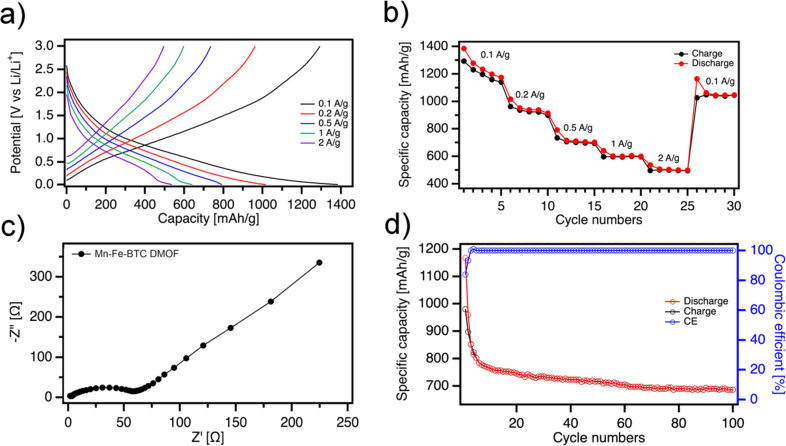
(a) Galvanostatic charge–discharge voltage profiles, (b) specific capacity at different current densities of Mn-Fe-BTC DMOF (c) EIS analysis and (d) cyclic performances.

## Conclusions

In summary, a straightforward hydrothermal method has been used to synthesize a porous dual metal–organic framework. Mn-Fe-BTC DMOF exhibits a high specific surface area (1045 m^2^ g^−1^), significant lithium storage capacity (specific capacity 1385 mA h g^−1^ at 0.1 A g^−1^), low charge transfer resistance (45.33 Ω), and robust long-term cyclability (specific capacity of 687 mA h g^−1^ after 100 charge/discharge cycles), attributed to its stable structure reinforced by π–π interactions. The findings suggest that Mn-Fe-BTC DMOF is a promising candidate for enhancing the efficiency and stability of LMBs. Our approach provides a rational strategy for designing functional porous materials for energy storage applications and can be extended to other metal ions and organic linkers, as well as to a multitude of promising applications.

## Conflicts of interest

There are no conflicts to declare.

## Supplementary Material

NA-006-D4NA00600C-s001

## Data Availability

Data for this article, including raw data for BET, FTIR, HR-TEM, SEM, STEM, TGA, XPS, XRD as well as electrochemical data (GCDs, EIS and CV) are available at Seafile Unistra Repository at https://seafile.unistra.fr/d/d2d6b6e065b1469f8f29/. Once the article will be accepted for print all the data will be made available (full open access) at the French National Open Access database – HALHAL Open Archives System (https://hal.science).
