# Supplementary material for: Mn–Fe dual metal–organic framework based on trimesic acid as a high-performance electrode for lithium metal batteries
Source: Nanoscale Adv. 2024 Sep 24;6(21):5301–5. doi: 10.1039/d4na00600c (PMC11441468; doi:10.1039/d4na00600c)
Supplement: NA-006-D4NA00600C-s001 [file NA-006-D4NA00600C-s001.pdf]

Supplementary Information (SI) for Nanoscale Advances.
This journal is © The Royal Society of Chemistry 2024

Supplementary Information

Mn-Fe dual metal–organic framework based on trimesic acid as high-performance electrode for lithium metal batteries

Saira Sarwar,[‡a] Verónica Montes-García,[‡b] Maria Stachowiak,[a, d] Tomasz Chudziak,[a, d] Wojciech Kukułka,[a] Cataldo Valentini,[a,d] Krzysztof Karoń,[c] Dawid Pakulski,*[a] Artur Ciesielski*[a,]

[a] Centre for Advanced Technologies, Adam Mickiewicz University, Uniwersytetu Poznańskiego 10, Poznań, 61-614, Poland. E-mail: dawid.pakulski@amu.edu.pl
[b] Université de Strasbourg, CNRS, ISIS 8 allée Gaspard Monge, Strasbourg 67000, France. E-mail: ciesielski@unistra.fr
[c] Faculty of Chemistry, Silesian University of Technology, Strzody 9, 44-100 Gliwice, Poland
[d] Faculty of Chemistry, Adam Mickiewicz University, Uniwersytetu Poznańskiego 8, Poznań 61-614, Poland

Table of Contents

Section A. Material characterization ... 2
Electrochemical Measurements ... 2
Electrode Preparation ... 2
Electrochemical calculations ... 3
Synthesis of Mn-Fe-BTC DMOF ... 3
Section B. Physical characterization ... 4
Raman ... 4
FTIR ... 4
XPS ... 5
TGA ... 5
SEM ... 6
CV ... 6
GCD ... 7
EIS ... 8
Post mortem analysis ... 8
State of the art ... 12
References ... 13

Section A. Material characterization

The following materials were procured for the study: manganese (II) chloride tetrahydrate, iron (III) chloride hexahydrate, benzene-1,3,5-tricarboxylic acid (BTC), lithium hexafluorophosphate in solution of ethylene carbonate (EC)-diethyl carbonate (DEC)-ethyl methyl carbonate (EMC) (1:1:1; vol), separators in the form of Whatman® glass microfiber filters, binder poly(vinylidene fluoride) (PVDF), and 1-methyl-2-pyrrolidinone (NMP) were all sourced from Merck. Conductive Carbon Black Super P (H30253) was obtained from Alfa Aesar.

The powder X-ray diffraction (PXRD) patterns were obtained using a Bruker AXS D8 Advance X-ray diffractometer with Cu Kα radiation (λ = 1.5405 Å) over a 2θ range from 6 ° to 60 °. The specific surface area and total pore volume were measured using a ASAP 2050 (Micromeritics) surface area and porosity analyzer. The sample was degassed at 100 °C for 12 hours under vacuum before analysis. A PerkinElmer TGA 8000 thermogravimetric analyzer was used to perform thermogravimetric analysis (TGA) over the temperature range from 25 to 800 °C under a N_2 atmosphere at a heating rate of 10 °C min^{-1}. Fourier-transform infrared (FTIR) spectra were recorded on a PerkinElmer Spectrum Two IR spectrometer using an attenuated total reflectance (ATR) mode within the range of 500-3500 cm^{-1}. Raman spectra were obtained using a Renishaw InVia Reflex system. The spectrograph used a high-resolution grating (1200 grooves cm^{-1}) with additional bandpass filter optics, a confocal microscope, and a 2D-CCD camera. The excitation was carried out using a 785 nm laser excitation beam, with a 100× objective, 0.02 mW maximum power and 10 s acquisition time The chemical valence states were determined using a Thermo Scientific KAlpha X-ray photoelectron spectrometer (XPS) with a mono-chromatized Al Ka anode as the X-ray source (1.486 eV) at a vacuum level of 10^{-8}–10^{-9} mbar in the main chamber. The spot size of the X-ray beam was fixed at 400 µm.

Electrochemical Measurements

The electrochemical performance of the as-prepared Mn-Fe-BTC DMOF was assessed in a two-electrode system. The galvanostatic charge/discharge (GCD) measurements were performed using a Neware battery test system and recorded in the voltage range of 0-3.0 V at current densities ranging from 0.1 to 2 A/g. The cyclic voltammetry (CV) analysis was performed using the Biologic MPG-2 instrument with scan rates of 0.5 and 0.1mV/s. Electrochemical impedance spectroscopy (EIS) was conducted with an amplitude of 10 mV and a frequency range of 100 kHz to 10 MHz. All the electrochemical analyses were performed at room temperature

Electrode Preparation

The cathode was prepared by mixing the Mn-Fe-BTC DMOF (75 % wt), PVDF binder (15 % wt), and conductive carbon black (10 % wt) in NMP. The slurry was evenly spread over Cu foil, creating a 200 µm-thick layer using a doctor blade. The electrodes were heated at 120 ºC in a vacuum oven overnight. Afterward, 18 mm discs were cut and used as cathodes. The assembling process was performed in a glovebox under a controlled argon (Ar) atmosphere, ensuring that the concentration of O_2 and H_2O remained below 0.1 ppm. The coin cell employed a Li-foil as an

anode, along with a glass microfiber paper as a separator. The electrolyte consisted of a solution of 1 M $LiPF_6$ in EC–DEC–EMC (1 : 1 : 1 vol%).

Electrochemical calculations

Calculation of the specific capacities. The specific capacity was calculated using GCD with the following equation:

$$C = \frac{I \; x \; \Delta t}{3.6 \; x \; m} \tag{1}$$

where I (A) is the applied current, Δt (s) is the discharge time, and m (g) is the mass of active material on the cathode.

Synthesis of Mn-Fe-BTC DMOF

A hydrothermal method was used to synthesize manganese/iron - benzene-1,3,5-tricarboxylic acid (Mn-Fe-BTC DMOF) for Li metal batteries. On the one hand, a solution (Solution A) was prepared by dissolving 1.5 g of manganese chloride tetrahydrate ($MnCl_2$ $4H_2O$) and 0.5 g of iron chloride hexahydrate ($FeCl_3{\cdot}6H_2O$) into 60 mL of EtOH. On the other hand, a second solution (Solution B) was prepared 1 g of BTC in 20 mL of EtOH. The solution A was gradually added to the solution B under magnetic stirring. After the addition of solution A was completed, 0.2 g of NaOH was added. After that, the solution was carefully transferred into a stainless-steel autoclave and placed in an oven at a temperature of 180 ^{o}C for 48 hours. The sample was collected by centrifugation after the autoclave naturally cooled down. It was then washed multiple times with deionized water and ethanol, and finally dried at 80 ^{o}C for 24 hours in an oven. A pinkish powder was obtained with yield occurs 87 % with reference to BTC.

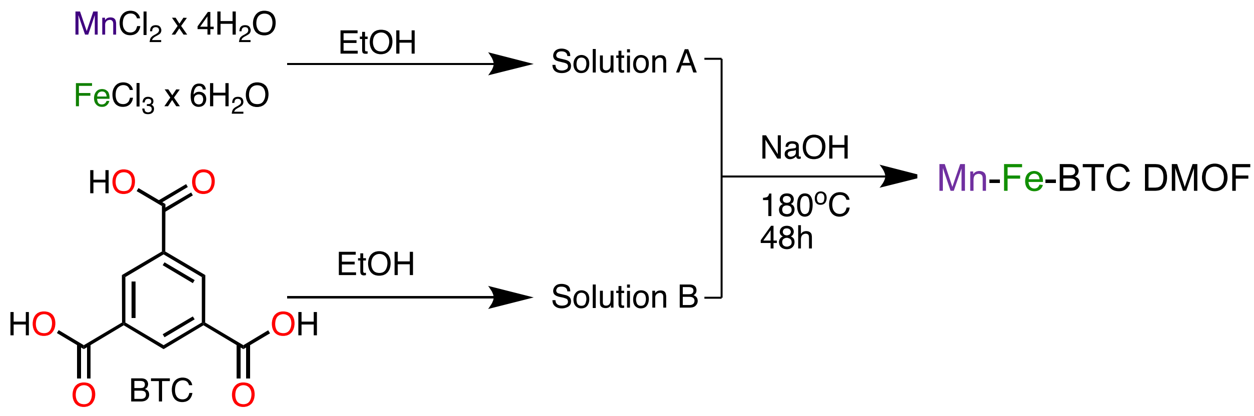

Fig. S1 Synthetic protocol of Mn-Fe-BTC DMOF.

Section B. Physical characterization

Raman

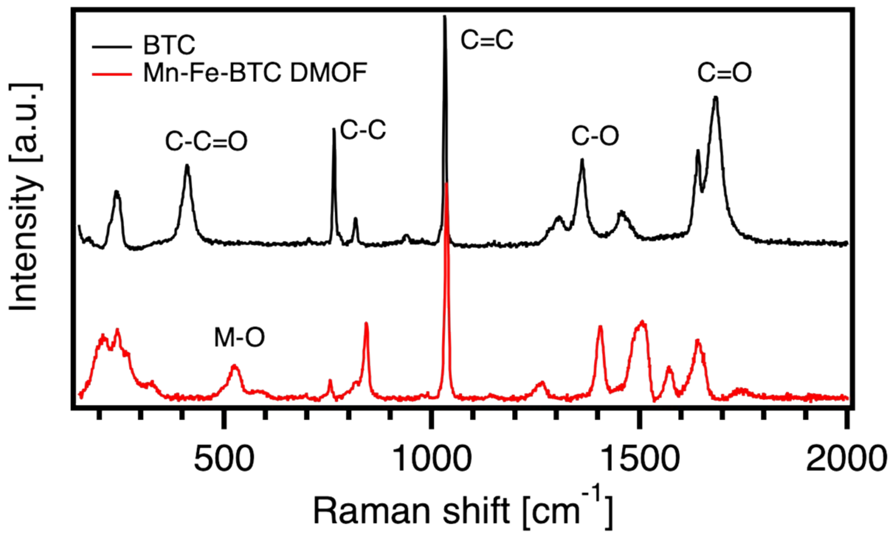

Fig. S2 Raman spectra of BTC (black line) and Mn-Fe-BTC (red line).

FTIR

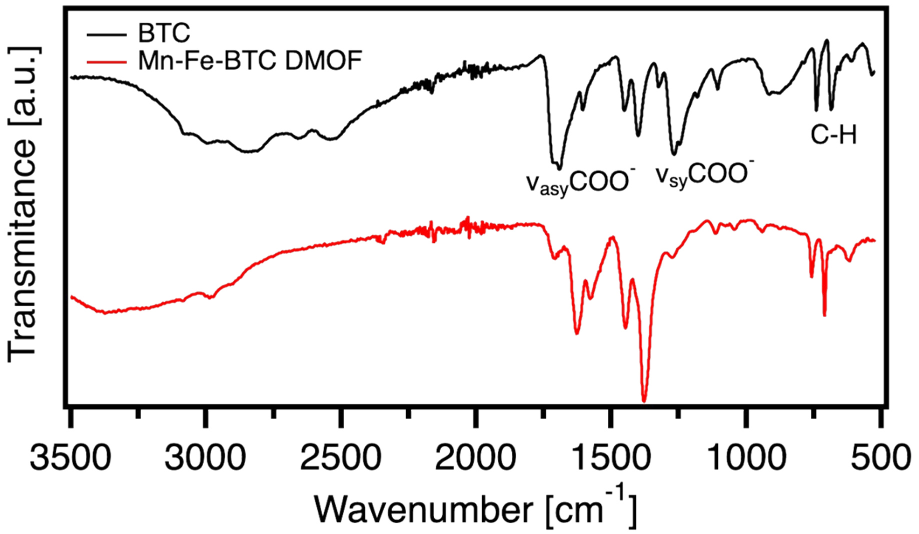

Fig. S3 FTIR spectra of BTC (black line) and Mn-Fe-BTC DMOF (red line).

XPS

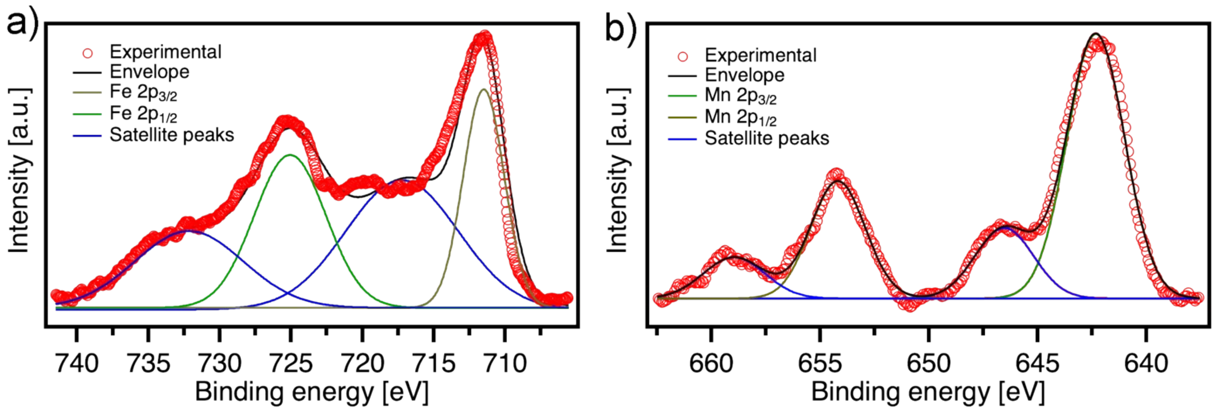

Fig. S4 XPS a) Fe2p and b) Mn2p spectra of Mn-Fe-BTC DMOF.

TGA

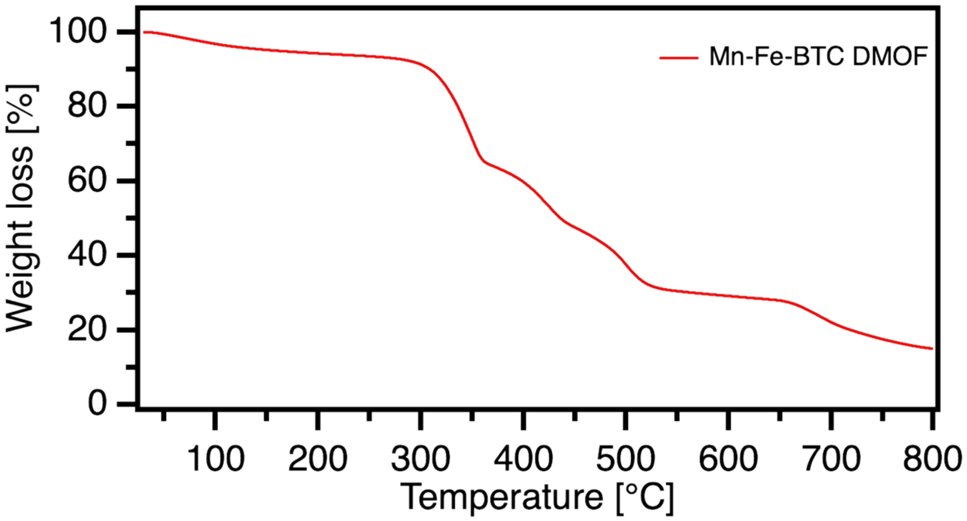

Fig. S5 TGA analysis of Mn-Fe-BTC DMOF.

SEM

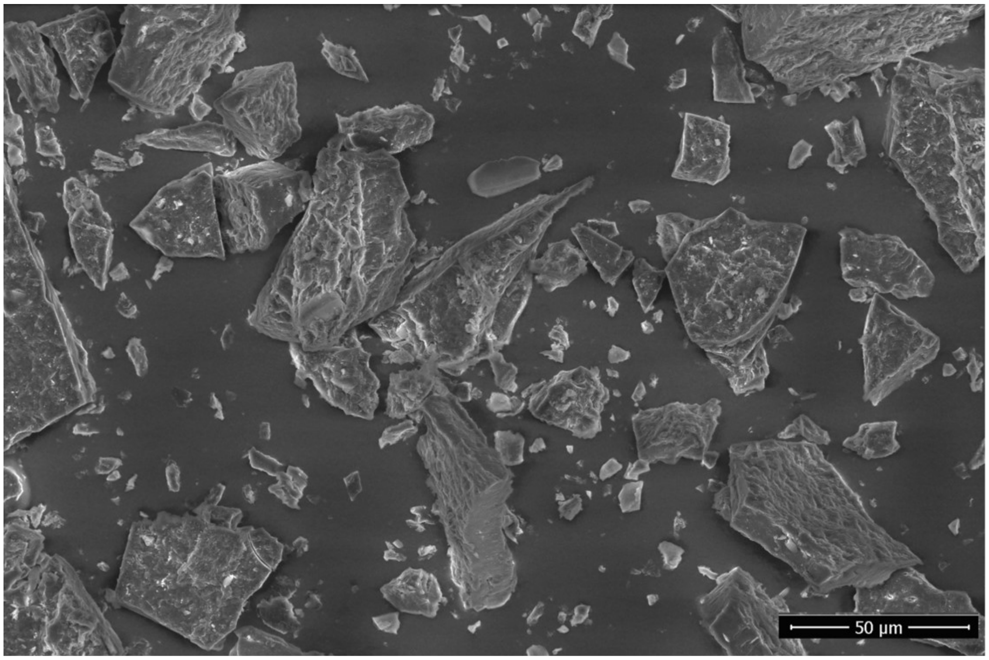

Fig. S6 Low-magnification SEM image of Mn-Fe-BTC DMOF.

CV

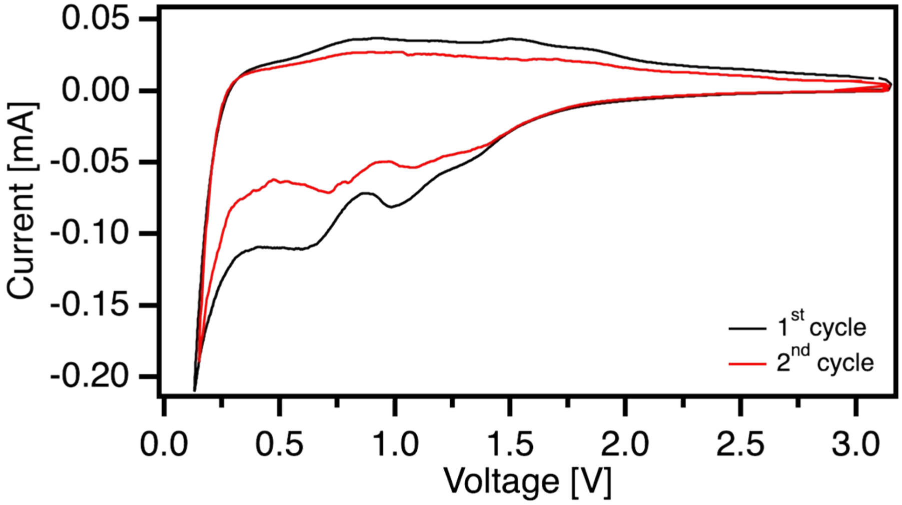

Fig. S7CV curves for Mn-Fe-BTC DMOF at 0.1 mV/s.

GCD

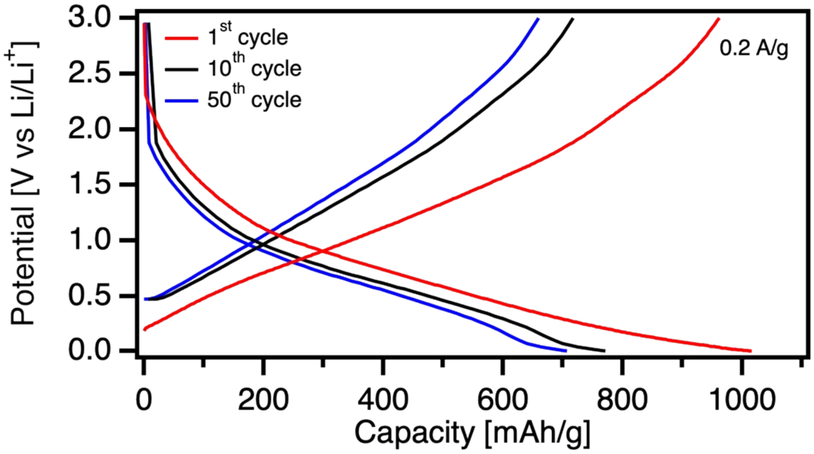

Fig. S8 Galvanostatic charge-discharge voltage profiles of Mn-Fe-BTC DMOF after 1, 10 and 50 cycles.

EIS

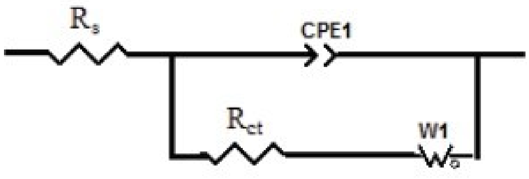

Fig. S9. The equivalent electric circuit model used for fitting the Nyquist plots.

Post mortem analysis

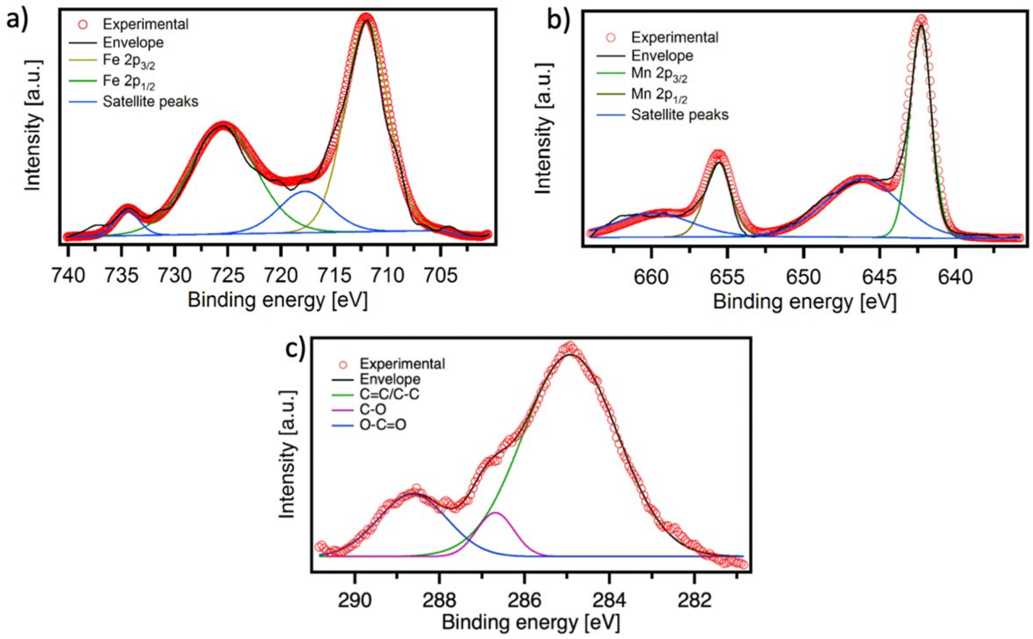

Fig. S10 XPS a) Fe2p, b) Mn2p, and c) C1s spectra of Mn-Fe-BTC DMOF electrode 100 charge/discharge cycles.

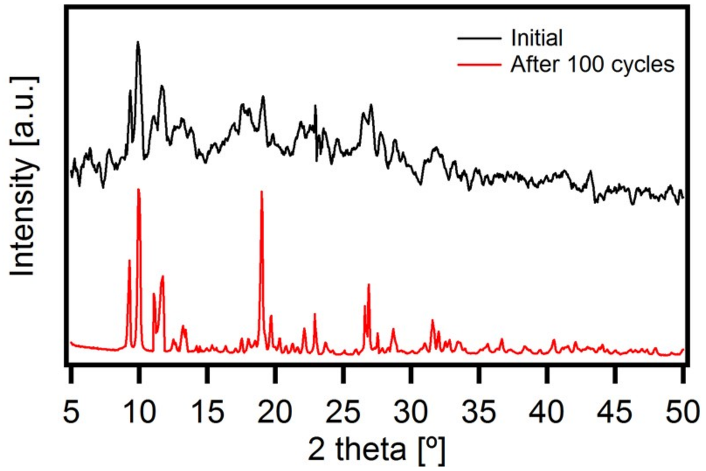

Fig. S11 PXRD spectra pristine Mn-Fe-BTC DMOF electrode (black) and after 100 charge/discharge cycles (red).

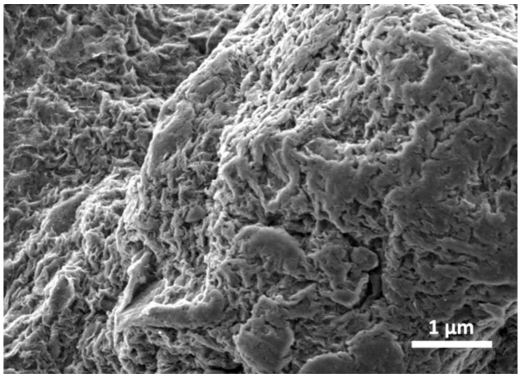

Fig. S12 SEM image of Mn-Fe-BTC DMOF electrode after 100 charge/discharge cycles.

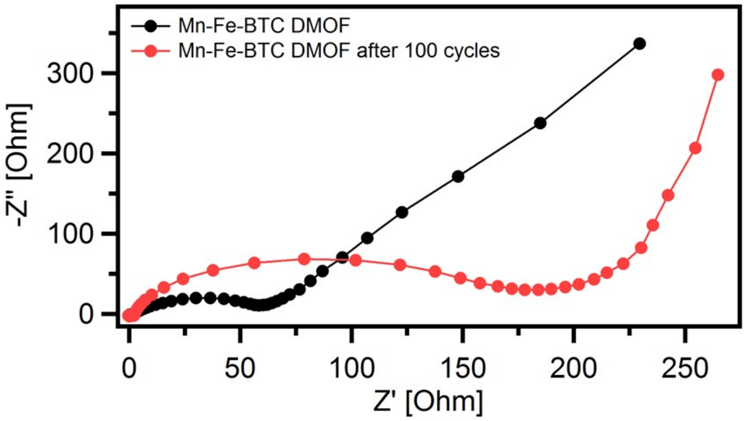

Fig. S13 EIS spectra of Mn-Fe-BTC DMOF after 1 cycle (black) and after 100 cycles (red).

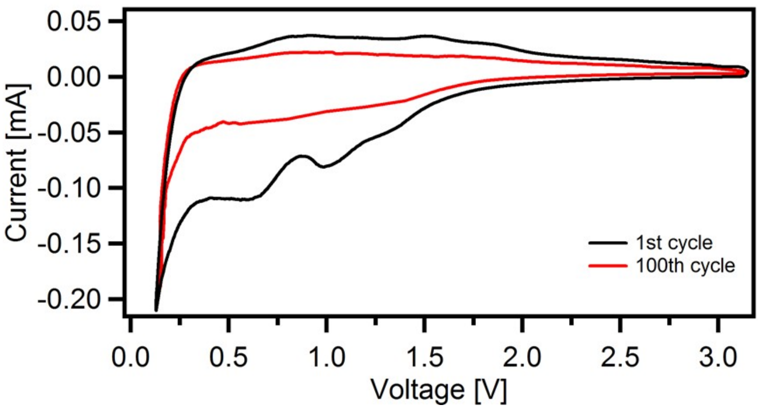

Fig. S14 CV curves for Mn-Fe-BTC DMOF at 0.1 mV/s after 1 cycle (black) and after 100 cycles (red).

State of the art

Tab. S1 Electrochemical performance of various Mn and Fe-based MOFs electrodes previously reported for LMBs.

Electrode	Surface area m^2/g	Electrolyte	Current density A/g	Specific capacity mAh/g	Cycle no.	Capacity retention mAh/g	Ref.
Mn-BDC	6.135	1 M $LiPF_6$ EC–DEC–EMC (1 : 1 : 1 vol%)	0.1	-	100	974	1
C/Fe- MOF (after calcination)	-	1 M $LiPF_6$ EC–DMC (1 : 1 vol%)	0.1	1124	50	975	2
Mn-BTC (after calcination)	24.9	1 M $LiPF_6$ EC–DEC (1 : 1 vol%)	0.1	630	60	582	3
NiFeMn-BTCA	263.23	1 M $LiPF_6$ EC–DMC–DEC (1 : 1 : 1 vol%)	0.1	802	100	624	4
$Mn_{1.8}Fe_{1.2}O_4$ MOF (after calcination)	124	1 M $LiPF_6$ EC–DEC (1 : 1 vol%)	0.2	2300	60	827	5
MnCo-T	9.15	1.0 M LiTFSI EC–DEC (1 : 1 vol%)	1	-	600	337	6
MnO/ZnO@C MOF (after calcination)	117.9	1 M $LiPF_6$ EC–DEC (1 : 1 vol%)	2	1396	1000	636	7
Ni/Mn-BTC (after calcination)	12.8	1 M $LiPF_6$ EC–DMC (1 : 2 vol%)	1.257	1049	400	258	8
Fe-BDC	-	1 M $LiPF_6$ EC–DMC (1 : 1 vol%)	0.03	280	40	175	9
Mn-BDC (after calcination)	4.12	1 M $LiPF_6$ EC–DEC–EMC (1 : 1 : 1 vol%)	0.1	961	100	653	10
H_8L-Fe-MOF	204	gel polymer electrolyte (GPE) membrane	0.05		50	275	11
**Mn-Fe-BTC DMOF**	**1045**	**1 M $LiPF_6$ EC–DEC–EMC (1 : 1 : 1 vol%).**	**0.1**	**1385**	**100**	**687**	**This work**

Abbreviations: *Mn-BDC*: Manganese-1,4-benzenedicarboxylate, *Fe-MOF* – Iron metal organic frameworks, *Mn-BTC* – Manganese Benzene-1,3,5-tricarboxylate, *NiFeMn-BTCA* - nickel, iron and manganese benzene-1,2,4,5-tetracarboxylate trimetallic organic framework , *MnCo-T* – manganese cobalt trimesic acid, , *Ni/Mn-BTC* – nickel, manganeze benzene-1,3,5-tricarboxylate, *Fe-BDC* - Iron 1,4-benzenedicarboxylate, *H_8L-Fe-MOF* – Iron ethene-1,1,2,2-tetrayltetrakis(benzene-4,1-diyl))tetraphosphonic acid.

References

1. H. Hu, X. Lou, C. Li, X. Hu, T. Li, Q. Chen, M. Shen and B. Hu, *New J. Chem.*, 2016, **40**, 9746-9752.
2. M. Li, W. Wang, M. Yang, F. Lv, L. Cao, Y. Tang, R. Sun and Z. Lu, *RSC Adv.*, 2015, **5**, 7356-7362.
3. F. Zheng, S. Xu, Z. Yin, Y. Zhang and L. Lu, *RSC Adv.*, 2016, **6**, 93532-93538.
4. D.-X. Yang, P.-F. Wang, H.-Y. Liu, Y.-H. Zhang, P.-P. Sun and F.-N. Shi, *J. Solid State Chem.*, 2022, **309**, 122947.
5. F. Zheng, D. Zhu, X. Shi and Q. Chen, *J. Mater. Chem. A*, 2015, **3**, 2815-2824.
6. W. Zheng, T. Hu, Y. Fang, L. Li and W. Yuan, *J. Solid State Chem.*, 2022, **306**, 122719.
7. F. Guo, H. Chen, Y. Chen, W. Zhang, Z. Li, Y. Xu, Y. Wang, J. Zhou and H. Zhang, *J. Alloys Compd.*, 2021, **852**, 156814.
8. S. Maiti, A. Pramanik, T. Dhawa, M. Sreemany and S. Mahanty, *Mater. Sci. Eng. B*, 2018, **229**, 27-36.
9. C. Ajpi, N. Leiva, A. Lundblad, G. Lindbergh and S. Cabrera, *J. Mol. Struct.*, 2023, **1272**, 134127.
10. J. Wang, Q. Yang and B. Wang, *Solid State Ion.*, 2023, **399**, 116273.
11. D. Chakraborty, T. Dam, A. Modak, K. K. Pant, B. K. Chandra, A. Majee, A. Ghosh and A. Bhaumik, *New J. Chem.*, 2021, **45**, 15458-15468.
